# Prevalence of and Factors Associated with Reproductive Tract Infections among Pregnant Women in Ten Communes in Nghe An Province, Vietnam

**DOI:** 10.2188/jea.15.163

**Published:** 2005-09-27

**Authors:** Aya Goto, Nguyen Quang Vinh, Pham Nghiem Minh, Kumiko Kato, Cao Thi Phi Nga, Le Thi Hoai Chung, Hoang Quoc Kieu, Le Thi Quynh Nga, Nguyen Ba Tan, Mayumi Katsube, Sumie Ishii, Seiji Yasumura

**Affiliations:** 1 Department of Public Health, Fukushima Medical University, School of Medicine.; 2 Tu Du Obstetrical and Gynecological Hospital.; 3 Hospital of University of Medicine and Pharmacy, Ho Chi Minh City.; 4 Department of Public Health, Yamagata University School of Medicine.; 5 Department of International Health, Harvard School of Public Health.; 6 Nghe An Maternal and Child Health / Family Planning Center.; 7 Training and Student Management Department, Nghe An Medical College.; 8 Department of Population and Family Health, Columbia University, Mailman School of Public Health.; 9 Japan International Cooperation Agency Nghe An Reproductive Health Project Office.; 10 Japanese Organization for International Cooperation in Family Planning.

**Keywords:** Reproductive Medicine, reproductive tract infection, Vietnam, Pregnancy

## Abstract

BACKGROUND: A community-based survey was conducted to investigate reproductive tract infections (RTIs) among pregnant women in Vietnam, where epidemiologic data on these infections are scarce.

METHODS: The focus of the study were: candidiasis, bacterial vaginosis, group B streptococcal infection, trichomoniasis, gonorrhea, syphilis, and hepatitis B. In addition to their prevalence, a generalized estimating equation was used to analyze infection-associated factors and diagnostic test analysis to examine the accuracy of currently performed presumptive clinical diagnoses.

RESULTS: Among 505 pregnant women in 10 communes, 182 (36%) had at least one infection with a wide regional variation in prevalence. The most prevalent infection was candidiasis (17%); sexually transmitted infections were rare except hepatitis B (10%); and the prevalence of bacterial vaginosis and group B streptococcal infections was 7% and 4%, respectively. Two factors were associated with the decreased risk of endogenous infections: a higher household assets score (odds ratio [OR] = 0.67) and condom use (OR = 0.15). Not living with a husband (OR = 1.55) was associated with an increased risk. For hepatitis B, three factors were associated with a decreased risk: employment by the government (OR = 0.26), higher education (OR = 0.18), and being older at the time of first sexual intercourse (OR = 0.58). Women’s self-reported symptoms and clinical findings had low positive predictive values. Only clinical findings from the vaginal wall showed both a sensitivity and specificity over 50%.

CONCLUSIONS: Suggested recommendations are: extensive application of microscopic diagnosis, prevention of hepatitis B transmission, and addressing the issues of regional differences in the prevalence of RTI and of less wealthy people.

The World Health Organization (WHO) estimated that 340 million new cases of trichomoniasis, gonorrhea, syphilis and chlamydia occurred globally in 1999 in men and women between the age of 15 to 49 years.^1^ The largest number of new infections occurred in South and Southeast Asia. Vietnamese health care workers believe the prevalence of a reproductive tract infection (RTI) among women to be high and they are concerned about their spread due to widely practiced induced abortions and intrauterine device insertion.^2^^,^^3^^,^^4^ Likewise, a report from one Vietnamese province stated that women believed RTI is one of their common morbidities; and they perceived dirty water and bad personal hygiene to be the causes for these infections.^5^ Despite people’s concerns, accurate epidemiologic data on RTI are scarce and existing information yields a widely varying RTI prevalence that ranges from 20% to 70%.^3^^,^^4^^,^^6^^,^^7^^,^^8^ In addition to true regional differences, there remains a question regarding the appropriateness of diagnostic techniques used in some studies.^9^ Two studies reported the RTI prevalence to be about 70% among women of reproductive age living in rural areas.^3^^,^^6^ These reports had the advantage of a community-based design; but the diagnoses of RTI relied heavily on clinical findings. The broad categorization of RTI included clinically suspected pelvic inflammatory disease, atrophic vaginitis and cervicitis.

Nghe An Province is located in the north central coastal region of Vietnam. The province is predominantly rural, relatively resource-poor, and includes 19 districts. With a population of 2,858,300, it has a birth rate of 21.6 per 1000. A long-term reproductive health project was launched by the Japan International Cooperation Agency (JICA) in 1997. The project is community-based and the Nghe An Maternal and Child Health / Family Planning Center (MCH/FP Center) was assigned as a project counterpart. The project objective was to improve reproductive health care, focusing on safe and hygienic delivery at the commune level. RTI was listed as one of the main reproductive health issues when phase II of the project started in 2000. According to the outpatient record of the Nghe An MCH/FP Center in 2002, around 40% of the gynecological patients were diagnosed with vulvitis, vaginitis or cervicitis and treated without identifying the pathogens. The results from a situational analysis of the reproductive health services in Nghe An Province revealed that the RTI treatment given was based mainly on clinical symptoms.^10^ The information suggests a lack of proper laboratory techniques and standardized case management in the region.

RTI is an important contributor to maternal and perinatal morbidity and mortality. Due to maternal immunosuppression during pregnancy, a higher incidence is observed for a variety of infections. The long-term adverse effects of RTI on women and their children include miscarriages, premature delivery, congenital malformation, and neonatal infections;^11^ yet none of the previous surveys in Vietnam investigated RTI prevalence among pregnant women in the community. Because a community-based study requires close interaction with the community and exhaustive preparatory efforts,^9^ a prevalence study in developing countries often targets pregnant women attending antenatal checkups at central health care facilities. However, these women tend to either belong to a high social class or suffer from abnormal symptoms. Taking advantage of the strong connection between the JICA project and the community, we carried out a first-time community-based survey in Vietnam to examine the prevalence and risk factors of RTI among pregnant women. Laboratory diagnostic techniques were introduced and compared against women’s symptoms and clinical findings.

## METHODS

### Survey Regions and Subjects

This cross-sectional survey was conducted among pregnant women in 10 communes of 4 districts in Nghe An Province. All communes were in suburban areas, which facilitated the transportation of samples to the MCH/FP Center. In addition, commune health centers (CHCs) in these regions had a favorable relationship with the MCH/FP Center, with joint JICA activities, which helped smooth implementation of the survey. The total number of married women between 15 and 49 years of age at the time of the survey in the 10 communes was 11,025. Nghi Thuy was a seaside resort frequented by tourists, Nghi Thuan and Hung Tay were Christian villages, and Nam Dan and Nam Thanh were relatively far from the urban area.

To encourage all pregnant women in the survey regions to participate, we utilized the pregnancy registration system at CHC and the local information network. The CHCs, women’s union and people’s committee were informed first of the survey dates, followed by individual women. In Nghe An Province, pregnant women receive an antenatal checkup at a local CHC, which keeps a list of pregnant women. Each CHC was visited and a list of “registered pregnant women” who had had an antenatal checkup at the center was obtained one week prior to the survey. Leaflets announcing the survey were distributed by the CHC staff to the registered women. The women’s union and people’s committee also made an announcement of the survey and encouraged pregnant women, including those whose pregnancy was not yet been registered, to participate.

### Survey Procedures

The survey consisted of 4 sections: an ultrasound checkup, interview, gynecological examination and a blood test. All women who came to participate in the survey were first screened for pregnancy by ultrasound, which served as a strong incentive to participate. After pregnancy confirmation, oral consent was obtained and the names of the women and the survey staff who explained the survey were recorded on the consent form. Each woman was personally interviewed by a trained physician or midwife, using a structured questionnaire. A speculum examination was performed to take swabs from the posterior fornix of the vagina and endocervix; and blood samples were taken. Interviews and examinations took place at the CHCs.

The pregnant women were informed of the test results at the local CHCs approximately 4 days after the survey. If a woman was found to have a treatable RTI, treatment was given free of charge. Medication for the woman’s partner was also dispensed if she was infected with trichomoniasis, syphilis or gonorrhea. A woman who was infected with hepatitis B was given a referral coupon for counseling regarding future risk reduction at the Preventive Medicine Center, a local health center specializing in care for hepatitis B.

### Questionnaire and Laboratory Tests

The questionnaire consisted of 30 questions on socioeconomic status, medical and reproductive health history, health behavior, and RTI symptoms. It was first developed in English, translated into Vietnamese, and further revised into the local dialect. Questions were asked on the presence of pruritus, sores or pain, dysuria and abnormal discharge as RTI symptoms. Household economic status was measured based on the presence of six household assets: access to electricity, a bicycle, a motorbike, availability of running water, a television, and a telephone.^12^^,^^13^ For personal hygiene, questions were included on “genital washing,” which is a local term mainly meaning vulvo-perineal washing and douching.

The seven target infections were candidiasis, bacterial vaginosis, group B streptococcal infection, trichomoniasis, gonorrhea, syphilis, and hepatitis B. The first three are classified as endogenous infections that result from an overgrowth of organisms normally present in the vagina. The other four are classified as sexually transmitted infections that are caused by organisms transmitted through sexual activity with an infected partner. Using vaginal wet mounts and gram stain microscopy, candidiasis, bacterial vaginosis and trichomoniasis were diagnosed. The Nugent criteria were applied to classify bacterial vaginosis.^14^ Bacterial cultures of the vaginal secretion were used to detect group B streptococcal infections and those from the cervical secretion to detect gonorrhea. Serum samples were evaluated for syphilis reactivity with the rapid plasma reagin test, which was confirmed with the Treponema pallidum hemagglutination test. For hepatitis B, samples were tested for HBsAg, using a quick test with confirmation by an enzyme-linked immunosorbent assay. The results were recorded on standard forms. All examination and laboratory tests were performed by trained staff from the MCH/FP Center or a laboratory at the Preventive Medicine Center. Wet mount microscopy was performed at the CHCs; gram stain microscopy, bacterial cultures, syphilis tests and hepatitis B quick tests at the MCH/FP Center; and hepatitis B ELISA test at the Preventive Medicine Center. Clinicians and laboratory technicians were trained prior to the survey, as noted below.

### Statistical Analysis

All information was entered into a database, using Access^®^(Microsoft Corporation, Redmond, WA) and analyzed with the aid of the software STATA^®^ version 8 for Windows (Stata Corporation, College Station, TX). First, for the analysis of the factors related to endogenous infections and hepatitis B, a population-averaged model of a generalized estimating equation was employed, adjusting for correlation among communes. The Huber-White sandwich estimator of the variance was used, with consideration given to spatial dependence. Nineteen variables were examined: sociodemographic information (age, residence in the commune all their lives, years of marriage, living with husband, religion, occupation, education, and household assets score); health behavior (health care seeking behavior when RTI symptoms were noted, husband’s past RTI symptoms, past contraceptive use, prior pregnancies, past abortion, and age at first sexual intercourse); hygienic practices (frequency of genital washing and type of water and material used); and data on current pregnancy (gestational week and frequency of antenatal care). Second, the woman’s self-reported symptoms and clinicians’ findings were compared with the laboratory diagnoses and the sensitivity, specificity, and positive predictive values were calculated.

### Preparatory Training and Pilot Study

Prior to the main survey, considerable efforts were made to enhance the local capability to conduct the RTI survey. After assessing the training needs of the local health professionals, epidemiologic, clinical and laboratory training was carried out at the MCH/FP Center for one month (December 2002). Then a pilot study was conducted in April 2003 to ensure the survey’s feasibility in and acceptability by the local community. Through the preparatory training and a pilot study, standards were established for the way the gynecological examinations and the interviews by 3 clinicians and 5 interviewers were to be conducted. Furthermore, Japanese and local experts supervised the interviews, examinations and laboratory procedures.

### Ethical Considerations

The study was approved by the ethics committee of Fukushima Medical University. In addition, a local research committee, whose members consisted of representatives from Nghe An Provincial Health Service, Provincial Hospital, and MCH/FP Center, was organized and authorized the survey.

## RESULTS

The total number of pregnant women registered at the CHCs was 435 and 377 (86%) of these women made up the survey participants. Additionally, 188 women who had their pregnancy confirmed on the survey days also participated. Of the 565 survey participants, 60 who failed to supply data in one or more of the 4 survey sections were excluded.

The median age of the 505 pregnant women with complete data was 27 (Table 1). For 63% their occupation was farmer and for 66% their educational attainment level at most was secondary school. The median score of household assets was 3. Thirty-five percent was primigravida, and only 17% had experience with condom use. A half of the women used salt for genital washing. The proportion of women who had RTI symptoms was as high as 92%. The differences in commune characteristics were: proportion of farmers, 0 to 90%; women with an educational level of high school or higher, 11 to 62%; and women with 5-6 household assets, 2 to 31%.

**Table 1.** Basic characteristics of pregnant women.

	Median (min, max)	N (%)*
		(N=505)
Age (years)	27 (18, 44)	
24 and younger		176 (34.9)
25-29		191 (37.8)
30 and over		138 (27.3)

Residence in commune whole life		
Yes		426 (84.4)
No		77 (15.3)

Religion		
None		425 (84.2)
Buddhist		4 ( 0.8)
Christian		76 (15.1)

Occupation		
Housewife		27 ( 5.4)
Agriculture		317 (62.8)
Business		62 (12.3)
Government employee		71 (14.1)
Others		28 ( 5.5)

Education		
None to primary school		64 (12.7)
Secondary school		271 (53.7)
High school		101 (20.0)
Professional school		53 (10.5)
University		15 ( 3.0)

Household assets^†^ (0-6)	3 (1, 6)	
1-2		118 (23.4)
3-4		323 (64.0)
5-6		64 (12.7)

Total number of past pregnancy	1 (0, 7)	
0		178 (35.3)
1		165 (32.7)
2 or more		162 (32.1)

Ever used contraceptives^‡^		
Intrauterine device		218 (43.2)
Withdrawal		203 (40.2)
Rhythm method		102 (20.2)
Condom		85 (16.8)
Oral contraceptives		44 ( 8.7)
Injectables		14 ( 2.8)

Material used for genital washing^‡^		
Soap		133 (26.3)
Medical liquid		91 (18.0)
Salt		252 (49.9)
Leaves		39 ( 7.7)

Gestational age (weeks)	26 (5, 42)	
12 or less		69 (13.7)
13-27		208 (41.2)
28 or more		228 (45.2)

Total number of present self-reported reproductive tract infection symptoms^§^	1 (0, 4)	
0		40 ( 7.9)
1		338 (66.9)
2-3		125 (24.8)

* : Total numbers for some items do not add-up to the total number in the top row because of some missing information.

† : Electricity, bicycle, motorbike, running water, television, and telephone were the assets measured.

‡ : Total percentage does not add-up to 100 because this was a multiple answer question.

§ : Pruritus, sore or pain, dysuria and abnormal discharge were asked.

Among the 505 pregnant women, 182 (36%) had at least one infection, the prevalence varying from 19% in Nam Thanh to 54% in Hung Tan (Table 2). The frequency of co-infections was 19 (4%) among the women with at least one infection. The most prevalent infection was candidiasis (17%) followed by hepatitis B (10%), the prevalence of which was strikingly high in Nghi Thuy compared with the other communes. STI was rare except hepatitis B; seven cases of trichomoniasis and one case of gonorrhea were found but no incidence of syphilis was detected. The prevalence of bacterial vaginosis and group B streptococcal infection was 7% and 4%, respectively.

**Table 2.** Prevalence of reproductive tract infections in ten communes.

Infections	N(%)										

	Total	Nghi Thuy	Nghi Khanh	Nghi Thuan	Nghi Xa	Hung Thong	Hung Tan	Kim Lien	Hung Tay	Nam Dan	Nam Thanh
	(N = 505)	(N = 46)	(N = 35)	(N = 47)	(N = 36)	(N = 44)	(N = 26)	(N = 111)	(N = 45)	(N = 52)	(N = 63)
Any infection*	182 (36)	19 (41)	14 (40)	14 (30)	14 (39)	20 (46)	14 (54)	37 (33)	13 (29)	25 (48)	12 (19)
Endogenous infections	143 (28)	10 (22)	11 (31)	13 (28)	13 (36)	16 (36)	11 (42)	25 (23)	10 (22)	18 (35)	11 (17)
Candidiasis	86 (17)	5 (11)	6 (17)	8 (17)	8 (22)	12 (27)	9 (35)	14 (13)	9 (20)	11 (21)	4 ( 6)
Bacterial vaginosis	35 ( 7)	2 ( 4)	4 (11)	4 ( 9)	2 ( 6)	3 ( 7)	2 ( 8)	8 ( 7)	1 ( 2)	5 (10)	4 ( 6)
Group B streptococcal infection	22 ( 4)	3 ( 7)	1 ( 3)	3 ( 6)	3 ( 8)	1 ( 3)	1 ( 4)	5 ( 5)	0 ( 0)	2 ( 4)	3 ( 5)
Sexually transmitted infections	56 (11)	13 (28)	4 (11)	2 ( 4)	2 ( 6)	5 (11)	3 (12)	13 (12)	3 ( 7)	9 (17)	2 ( 3)
Trichomoniasis	7 ( 1)	2 ( 4)	0 ( 0)	0 ( 0)	0 ( 0)	0 ( 0)	0 ( 0)	3 ( 3)	1 ( 2)	0 ( 0)	1 ( 2)
Gonorrhea	1 ( 0)	0 ( 0)	0 ( 0)	0 ( 0)	0 ( 0)	1 ( 3)	0 ( 0)	0 ( 0)	0 ( 0)	0 ( 0)	0 ( 0)
Syphilis	0 ( 0)	0 ( 0)	0 ( 0)	0 ( 0)	0 ( 0)	0 ( 0)	0 ( 0)	0 ( 0)	0 ( 0)	0 ( 0)	0 ( 0)
Hepatitis B	50 (10)	12 (26)	4 (11)	2 ( 4)	2 ( 6)	4 ( 9)	3 (12)	10 ( 9)	3 ( 7)	9 (17)	1 ( 2)

* “Any infection” indicates the number and proportion of women infected with at least one of the seven target infections.

Tables 3 and 4 show the factors associated with endogenous infections and hepatitis B in the generalized estimating equation model. Having a score of 5-6 household assets (odds ratio [OR] = 0.67, 95% confidence interval [CI] = 0.43-1.05) and condom use only (OR = 0.15, 95% CI = 0.03-0.88) were associated with a decreased risk of endogenous infections. On the other hand, not living with their husband was associated with an increased risk (OR = 1.55, 95% CI = 1.12-2.13). For hepatitis B, three factors were associated with a decreased risk of infection: being employed by the government (OR = 0.26, 95% CI = 0.06-1.05); graduate of a professional school or university (OR = 0.18, 95% CI = 0.04-0.95); and first sexual intercourse at the age of 20 or later (OR = 0.58, 95% CI = 0.32-1.07).

**Table 3.** Factors associated with endogenous infections among pregnant women.

	[N (%)]^†^		Odds ratio (95%CI)

	Infected	Not infected	
	(N=138)	(N=367)	
Living with husband			
Yes	109 (80.2)	317 (86.6)	1.00 (reference)
No	27 (19.9)	49 (13.4)	1.55 (1.12-2.13)*

Household assets ^‡^ (0-6)			
1-4	124 (89.9)	317 (86.4)	1.00 (reference)
5-6	14 (10.1)	50 (13.6)	0.67 (0.43-1.05)^#^

Past contraceptive use			
Never used	52 (37.7)	108 (29.4)	1.00 (reference)
Condom only	1 ( 0.7)	14 ( 3.8)	0.15 (0.03-0.88)*
Other methods ^§^	85 (61.6)	245 (66.8)	0.72 (0.47-1.11)

# p<0.1, * p<0.05. Population-averaged model of a generalized estimating equation adjusting for possible correlation between communes was employed.

† : Total numbers for some items do not add-up to the total number in the top row because of some missing information.

‡ : Electricity, bicycle, motorbike, running water, television, and telephone were the assets measured.

§ : “Other methods” comprise intrauterine device, withdrawal, rhythm method, oral contraceptives, injectables, and combined usage of these methods and condoms.

CI: confidence interval.

**Table 4.** Factors associated with hepatitis B among pregnant women.

	[N (%)]^†^		Odds ratio (95%CI)

	Infected	Not infected	
	(N=50)	(N=455)	
Occupation		
Agriculture	28 (56.0)	289 (63.5)	1.00 (reference)
Business	13 (26.0)	49 (10.8)	2.25 (0.86-5.89)
Government employee	2 ( 4.0)	69 (15.2)	0.26 (0.06-1.05)^#^
Housewife / others	7 (14.0)	48(10.6)	1.26 (0.62-2.56)

Education			
None to primary school	8 (16.0)	56 (12.3)	1.00 (reference)
Secondary school	30 (60.0)	241 (53.1)	0.89 (0.43-1.85)
High school	10 (20.0)	91 (20.0)	0.76 (0.27-2.18)
Professional school / University	2 ( 4.0)	66 (14.5)	0.18 (0.04-0.95)*

Age at first sexual intercourse (years)			
younger than 20	16 (32.0)	107 (23.6)	1.00 (reference)
20 and over	34 (68.0)	346 (76.4)	0.58 (0.32-1.07)^#^

# p<0.1, * p<0.05. Population-averaged model of a generalized estimating equation adjusting for possible correlation between communes was employed.

† : Total numbers for some items do not add-up to the total number in the top row because of some missing information.

Compared with the laboratory diagnosis, the women’s self-reported symptoms and clinical findings had low positive predictive values (Table 5). Only the clinical findings on the vaginal wall had both a sensitivity and specificity over 50%.

**Table 5.** Comparison of laboratory diagnosis with women’s symptoms and clinical diagnosis.

	Laboratory diagnosis			Total (N)	Sensitivity (%)	Specificity (%)
	[N (%)]*					

	Infected^†^	Not infected				
Women’s self-reported symptoms						
Pruritus						
No	110 (26.1)	311 (73.9)		421		
Yes	34 (*41.0*)	49 (59.0)		83	23.6	86.4

Sore or pain						
No	127 (27.9)	329 (72.1)		456		
Yes	17 (*35.4*)	31 (64.6)		48	11.8	91.4

Dysuria						
No	127 (28.2)	323 (71.8)		450		
Yes	17 (*32.1*)	36 (67.9)		53	11.8	90.0

Abnormal discharge						
No	10 (20.0)	40 (80.0)		50		
Yes	134 (*29.5*)	320 (70.5)		454	93.1	11.1

Have at least one symptom						
No	5 (12.5)	35 (87.5)		40		
Yes	139 (*30.0*)	324 (70.0)		463	96.5	10.0

Physician’s findings						
Vaginal wall ^†^						
Normal	67 (25.5)	197 (74.9)		263		
Abnormal	77 (*32.2*)	162 (67.8)		239	53.3	54.9

Discharge						
Normal	43 (27.0)	116 (73.0)		159		
Abnormal	101 (*29.4*)	242 (70.6)		343	70.1	32.4

Cervix						
Normal	136 (28.9)	334 (71.1)		470		
Abnormal	8 (*25.0*)	24 (75.0)		32	5.6	93.3

* : Percentages in italics are positive predictive values.

† : Infections in this analysis comprise trichomoniasis, candidiasis, bacterial vaginosis, group B streptococcal infection, gonorrhea and syphilis.

‡ : Clinical findings of vaginal wall were recorded as “normal”, “inflammation” or “others”.

The latter two were classified as abnormal.

## DISCUSSION

This community-based survey, with laboratory diagnosis to define RTI, provided information on RTI among pregnant women in communes in Vietnam for the first time. Endogenous infections were found to be most prevalent among pregnant women in the target region, as in previous studies conducted among non-pregnant Vietnamese women.^4^^,^^7^^,^^8^ Candidiasis had the highest prevalence, which was comparable to that previously reported in developed countries. In a multi-center cohort study among pregnant women in the United States, the prevalence of moderate to heavy candida colonization was 10%.^15^ During pregnancy, increased levels of transudation from the vaginal wall, glycogen in the vaginal secretions, a progesterone level and impaired immunity predispose one to candidiasis. Fortunately, this prevalent infection does not cause serious negative consequences to one’s pregnancy,^15^ but a current presumptive diagnosis could lead to an over-prescription of antibiotics and subsequent unnecessary morbidity. This can be addressed by introducing appropriate diagnostic techniques, as described below.

The prevalence of bacterial vaginosis reported in a previous multi-center survey in seven countries that used the same diagnostic techniques varied from 5.8% in the United States to 24.4% in Zimbabwe.^16^ With regard to the group B streptococcal infection, a study from Peru reported a prevalence of 6%; and another study in the United States reported an even higher 18.6%.^17^^,^^18^ Although the prevalence of the two infections in Nghe An Province was relatively low when compared with the ones reported in the aforementioned studies, these two are known as strong risk factors for preterm delivery and neonatal infection.^19^^,^^20^ The reviews by the Cochrane Collaboration and the WHO recommend that high-risk women with a history of preterm delivery be screened for bacterial vaginosis.^21^^,^^22^ Again on the group B streptococcal infection, the Center for Disease Control and Prevention of the United States recommends universal culture-based screening at the 35-37th week of gestation.^23^ Further, one previous randomized controlled trial reported a reduction in preterm births by integrating a simple microscopic detection procedure for bacterial vaginosis, candidiasis, and trichomoniasis into antenatal care.^24^ In Nghe An Province, a gynecological examination is not a routine procedure in antenatal care and universal prenatal screening is not realistic in a resource-poor setting; but screening women at high risk may be feasible, especially in urban areas. The risk of a preterm birth is reported to be rather high (12%) in Vietnam.^25^ Follow-up studies of the obstetrical influence on these infections are needed to explore the potential impact of introducing a screening program.

The STI prevalence was extremely low compared with that in African countries,^26^^,^^27^ except for hepatitis B. The main transmission route of hepatitis B in an endemic area is exposure early in life through vertical or horizontal transmission.^28^ There is a hepatitis B immunization program for all babies born in Nghe An Province, but local health care workers report a shortage of vaccines and little recognition of how important the program is among parents. Moreover, an association with age at the time of first sexual intercourse, the only item related to sexual behavior in our questionnaire, and a wide inter-commune variation may suggest sexual and iatrogenic components in the spread of infection. The urgent issues to be considered are the protection of the newborns from vertical transmission, prevention of further sexual transmission in the reproductive age group, and implementation of infection control measures at health care institutions.

Despite local concerns and several earlier studies reporting a high RTI prevalence in Vietnam, we found the prevalence of STI, except hepatitis B, to be extremely low; and endogenous infections to be moderate, as discussed above. First, the most probable explanation for this is the laboratory test-based diagnosis, which is one of the strengths of the present study. Hue study, in which the most likely diagnostic methods were applied, reported a relatively low RTI prevalence among patients at a local MCH/FP Center.^7^ Second, another possible explanation is the unique characteristics of the target population in our study. Pregnant women are characterized as a low-risk population for STI; but as already noted, they are prone to be infected with candidiasis. The third possible explanation, particularly in regard to the low STI prevalence, is the low risk sexual behavior in Vietnam. One study in the northern part of Vietnam revealed that only a small proportion of subjects aged 15-49 years had a sexual relationship with someone other than their regular partners or with commercial sex workers.^29^ However, the spread of HIV has shifted toward a younger population: the public should be made aware of the tendency toward an increase in the sexual transmission of STI and HIV due to increasing sexual activity among this population segment.^29^^,^^30^

Interestingly, condom use was proven to be a protective factor for endogenous infections. There is growing evidence that shows a possible sexual component in the occurrence of bacterial vaginosis, candidiasis and group B streptococcal infections. Similar to our study, condom use showed a significant protective effect against bacterial vaginosis in some earlier studies.^31^^,^^32^ Others have reported that when the sexual partner of a patient with recurrent vaginal candidiasis is treated, the recurrence rate decreases, and that a group B streptococcal infection is associated with sexual activity.^33^^,^^34^ In the present study, only 17% had ever used condoms: so as the HIV epidemic in Vietnam continues to evolve rapidly,^30^ there is a need to promote condoms for the prevention of RTI and HIV/AIDS.

For a better understanding of gynecological morbidity, there is a need to investigate social determinants.^9^ We found an association between a higher household asset score and a decreased risk of endogenous infections; and a higher educational level and being a government employee, a respected occupation in a socialist society, with a decreased risk of hepatitis B. It is known that hepatitis B has a strong association with being in a low social class.^35^ For endogenous infections, one RTI survey among pregnant Bangladeshi women revealed an association between low socioeconomic status and bacterial vaginosis.^36^ Poverty is linked with other factors that are related to infections, including a lack of access to preventive and curative health services, low health literacy, unfavorable sanitation, and risky sexual behavior.^37^ An RTI prevention program targeted at the less wealthy population is recommended.

The Vietnamese women’s perceived causes of RTI are predominantly exposure to dirty water and poor personal hygiene.^5^ Although the practice of “genital washing” was not associated with RTI in the current survey, it was noted that half of the women used salt and some used leaves. In general, women of all ages should be advised to avoid vaginal douching. Further investigation is needed to understand the ways Vietnamese women practice “genital washing” and their belief in regard to douching practices.

In an earlier study in Hue,^7^ the investigator stated that symptoms and clinical findings were not good indicators of an RTI infection and could lead to over-diagnosis and over-treatment. One study on a low-income, low RTI prevalence situation in Bangladesh pointed out a likely problem of over-treatment due to syndromic management.^37^ Given the rapid increase in the drug supply since economic reform in 1986 (Doi Moi) and few regulations on its application, we recommend that laboratory diagnosis of common endogenous infections be promoted at health care institutions with laboratory facilities.^7^ Endogenous vaginal infections can be diagnosed by a simple microscopic test. Microscopes are available at local hospitals and other health care institutions and this study demonstrated that local laboratory staff and physicians can diagnose the infections if adequate training is given. The MCH/FP Center had already organized a three-day RTI diagnosis training session for gynecologists and laboratory technicians at district hospitals in Nghe An Province, a repetition of which is recommended.

To conduct prevention programs effectively, intra-province variations in prevalence should be considered. For example, Hung Tan needs to target endogenous infections and preventing hepatitis B should be the top priority in Nghi Thuy. Also, the pregnancy registration system should be reviewed. Surprising to local health policy makers, the registration system at CHC lacked data on a considerable number of pregnant women. In Vietnam, all health data are first collected at the CHC level and summarized for each district and province level, from which national statistics are compiled. Improved and accurate data collection at the CHC level is mandatory for health policy development and promotion activities. The JICA project gave support to ensuring the utilization of Home-Based Maternal Records (HBMR) for pregnant women and mothers to keep a record of their antenatal visits and child growth; and for providing an exact copy of the handbook for each woman at the level of the CHC. This system should be strengthened to improve the pregnancy registration system.

There are three major limitations in the current study. First, the results obtained from this cross-sectional study can only provide evidence of statistical associations between RTI and the factors that were investigated: it does not show cause-effect relationships. Second, because this was the very first RTI survey among pregnant women in the region, detailed questions on sexual behavior and questions to test a woman’s knowledge about RTI were not included in the questionnaire because of concern about their acceptance of the survey. The MCH/FP Center is planning to repeat a similar RTI survey to monitor the trend in RTI prevalence after implementing several preventive measures. These opportunities should be utilized to investigate further both women’s and men’s knowledge about reproductive health and related behavior. Third, not all of the laboratory tests that were done could be classified as optimal, considering the local laboratory capacity and the feasibility of repeating a similar survey by local staff in the future. It is expected that better diagnostic tests and new infections will be added in future surveys, provided that the laboratory techniques and facilities also improve.

In summary, endogenous infections were prevalent among pregnant women in Nghe An Province. The design and implementation of interventions to prevent RTI require a multifaceted approach. From a medical perspective, promotion and training in conducting simple microscopy tests to diagnose endogenous infections are recommended. Preventing vertical and horizontal transmission of hepatitis B is an urgent issue in some regions. Educational programs promoting condom use and improving people’s knowledge about RTI prevention are recommended. For the effective operation of prevention programs, intra-province variation in RTI prevalence should be addressed, pregnancy registration systems require improvement and less wealthy people need special attention.
